# A fatal case of Severe Fever with Thrombocytopenia Syndrome (SFTS): Early morphological clues in peripheral blood and lessons for clinical alert

**DOI:** 10.1016/j.idcr.2026.e02766

**Published:** 2026-09-11

**Authors:** Junma You, Zhiyong Feng, Hongsheng Zhang

**Affiliations:** a Department of Clinical Laboratory, Yichang Central People’s Hospital, Yichang, People's Republic of China; b The First College of Clinical Medical Science, China Three Gorges University, Yichang, People's Republic of China

**Keywords:** Blood picture, Peripheral blood morphology, Severe fever with thrombocytopenia syndrome

## Abstract

This report details the first rapid diagnosis of severe fever with thrombocytopenia syndrome (SFTS) at our institution using peripheral blood morphology and highlights its key hematological features for early detection. A 59-year-old male farmer was admitted with chills, fever, and diarrhea lasting 8 days. Diagnosis was confirmed using blood tests and next-generation sequencing. Within 24 h, lab tests revealed key diagnostic clues, including abnormal blood counts and distinctive cell morphology. Despite aggressive treatment, the patient's condition worsened, leading to clinical death. In summary, SFTS is a deadly infection with high mortality. In endemic areas, quick screening of suspected cases using peripheral blood morphology by knowledgeable lab staff can lead to early diagnosis and timely treatment, essential for better clinical outcomes.

## Highlights

• Neutrophil phagocytosis: a novel finding in SFTS.
• Green neutrophilic inclusions predict mortality in SFTS.
• Blood smear enables rapid SFTS diagnosis in endemic areas.
• Plasmacytoid lymphocytes and apoptosis indicate poor prognosis.

## Introduction

Severe fever with thrombocytopenia syndrome (SFTS) is a tick-borne hemorrhagic fever caused by the novel bunyavirus (SFTSV), first identified in China in 2007 [1]. The disease is characterized by high fever, thrombocytopenia, leukopenia, and gastrointestinal symptoms, and may progress to multi-organ failure and death in severe cases, with a case fatality rate of up to 30% [2,3]. SFTS is predominantly endemic in East Asian countries, including China, Japan, and South Korea, and its incidence has demonstrated a significant upward trend in recent years, raising increasing public health concerns [4]. Although the clinical and laboratory profiles of SFTS have been extensively documented, the systematic characterization of early peripheral blood morphological changes—readily obtainable upon hospital admission—remains inadequately addressed. Understanding these features is crucial for facilitating early screening, refining differential diagnosis, and guiding timely clinical intervention.

This analysis investigates the blood morphology of a 59-year-old male farmer with SFTSV infection, highlighting both common and new hematological findings within 24 h of admission.We identify unusual granulocyte forms as a new source of smudge cells and report neutrophil green bodies and cytoplasmic inclusions, previously unlinked to SFTS.We propose a morphological triad with potential prognostic value.By combining automated digital microscopy with expert review,this study emphasizes the importance of early detailed morphological analysis in enhancing diagnosis, understanding disease mechanisms,and assessing risk in SFTS.

## Case presentation

A 59-year-old male farmer presented to our institution following 8 days of chills, fever, and profuse watery diarrhea (>20 episodes/day), with an associated non-productive cough. He had received 3 days of targeted therapy at a local hospital, including ribavirin (antiviral) and ceftriaxone (antibiotic), alongside supportive care; however, fever persisted, and blood cell counts dropped progressively, prompting transfer for further evaluation.

On admission, laboratory studies revealed leukocytopenia (WBC 2.07 ×10⁹/L), normal hemoglobin (121 g/L; RBC 3.73 ×10 ¹²/L), and severe thrombocytopenia (PLT 12 ×10⁹/L). Peripheral blood smear review highlighted key findings: marked leukopenia with severe granulocytic left shift (1% metamyelocytes, 21% band neutrophils, 52% segmented neutrophils), accompanied by toxic changes (coarse granulation, vacuolization, Döhle bodies); 6% atypical lymphocytes; anisocytosis; scant platelets with large, immature forms; and numerous smudge cells (predominantly granulocytic). Collectively, these morphological features—consistent with a severe systemic infectious process—raised strong suspicion for novel bunyavirus infection, given the clinical context.

The attending physician, suspecting a severe viral infection, promptly ordered nucleic acid testing for the novel bunyavirus and transferred the patient to the intensive care unit(ICU). Confirmatory evidence was obtained the following day through whole-blood metagenomic next-generation sequencing (mNGS), which identified concurrent infections: novel bunyavirus (283091 reads; 99% confidence), Aspergillus flavus (2096 reads; 99% confidence), and Epstein-Barr virus (EBV; 101 reads; 99% confidence).

In the ICU, a comprehensive treatment regimen was implemented, encompassing aggressive anti-infective therapy, anti-shock measures, continuous renal replacement therapy (CRRT), and intra-aortic balloon pump (IABP) support. Although these interventions resulted in improvements, such as decreased inflammatory markers, increased platelet counts, and improvement of myocardial enzymes, liver function, and coagulation parameters, they were insufficient to counteract the progression of heart failure and pulmonary edema. By the fifth day of hospitalization, the patient's family decided to withdraw all life-sustaining treatments, and the patient was subsequently pronounced deceased.

## Discussion

A hematological evaluation conducted within 24 h of admission identified critical morphological characteristics associated with severe fever with thrombocytopenia syndrome (SFTS) in this case. This finding contributes to the advancement of current understanding by aligning with, and diverging from, established literature, particularly as discussed in Reference 5.

**Fig. 1:**
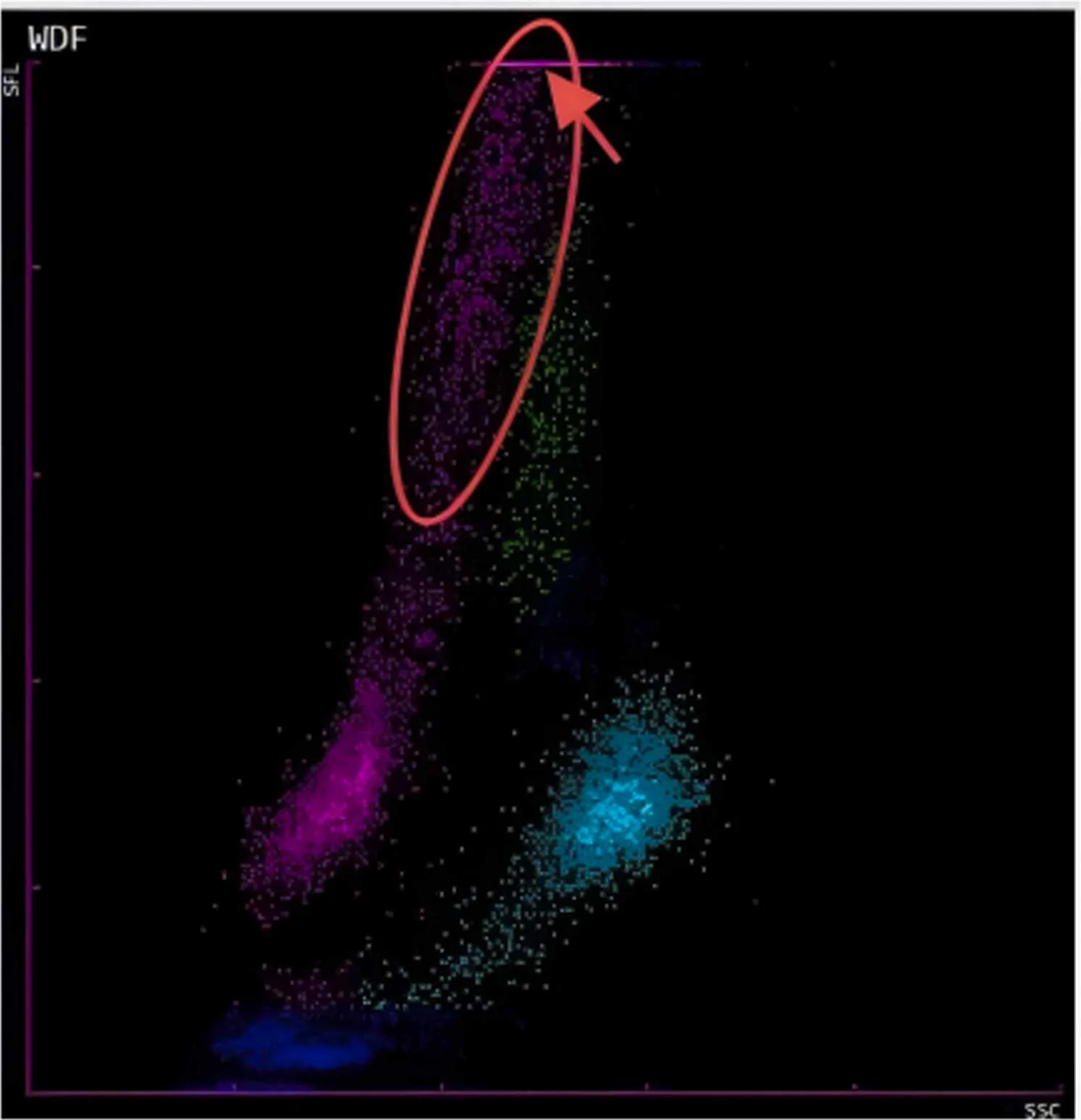
Blood test scattergram at admission shows numerous abnormal lymphocytes (circled) and a cluster of excessively large lymphocytes (arrow) exceeding coordinate boundaries.

**Fig. 2:**
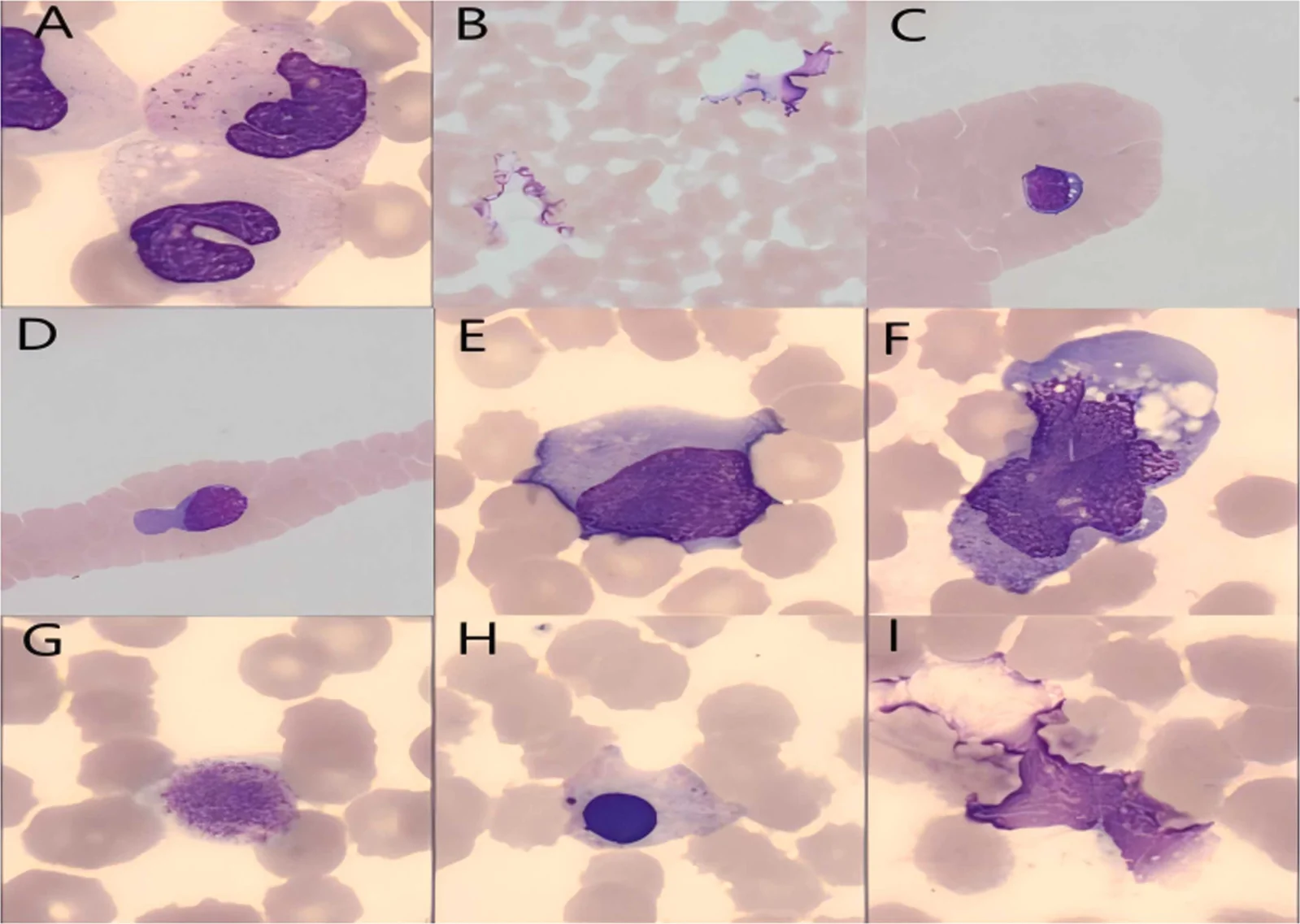
Peripheral blood Wright-Giemsa stain (×1000). A: Neutrophil with toxic granules, vacuoles, and Döhle bodies. B: Abnormal neutrophil with eccentric nucleus and pale cytoplasm. C-E: Plasmacytoid atypical lymphocytes. F: Activated monocyte with enlarged body, abundant cytoplasm, and vacuoles. G: Giant platelet. H: Apoptotic cell. I: Smudge cell.

The peripheral blood analysis revealed leukopenia, thrombocytopenia, and an abnormal white blood cell scattergram (Fig. 1), while erythroid parameters remained within normal limits. Morphological examination identified a pronounced granulocytic left shift, with 1% metamyelocytes and 21% band forms, accompanied by significant toxic changes such as coarse granulation, cytoplasmic vacuolization, and Döhle bodies (Fig. 2A), consistent with the primary feature reported in Reference 5. Notably, this case unveiled a previously unreported subpopulation of dysmorphic granulocytes, characterized by eccentric, irregular nuclei and hypogranular, pale cytoplasm (Fig. 2B), a phenotype observed across immature stages. Importantly, these abnormal granulocytes appeared to be the primary source of the abundant smudge cells (Fig. 2I), in contrast to the lymphoid origin proposed in Reference 5.

**Fig. 3:**
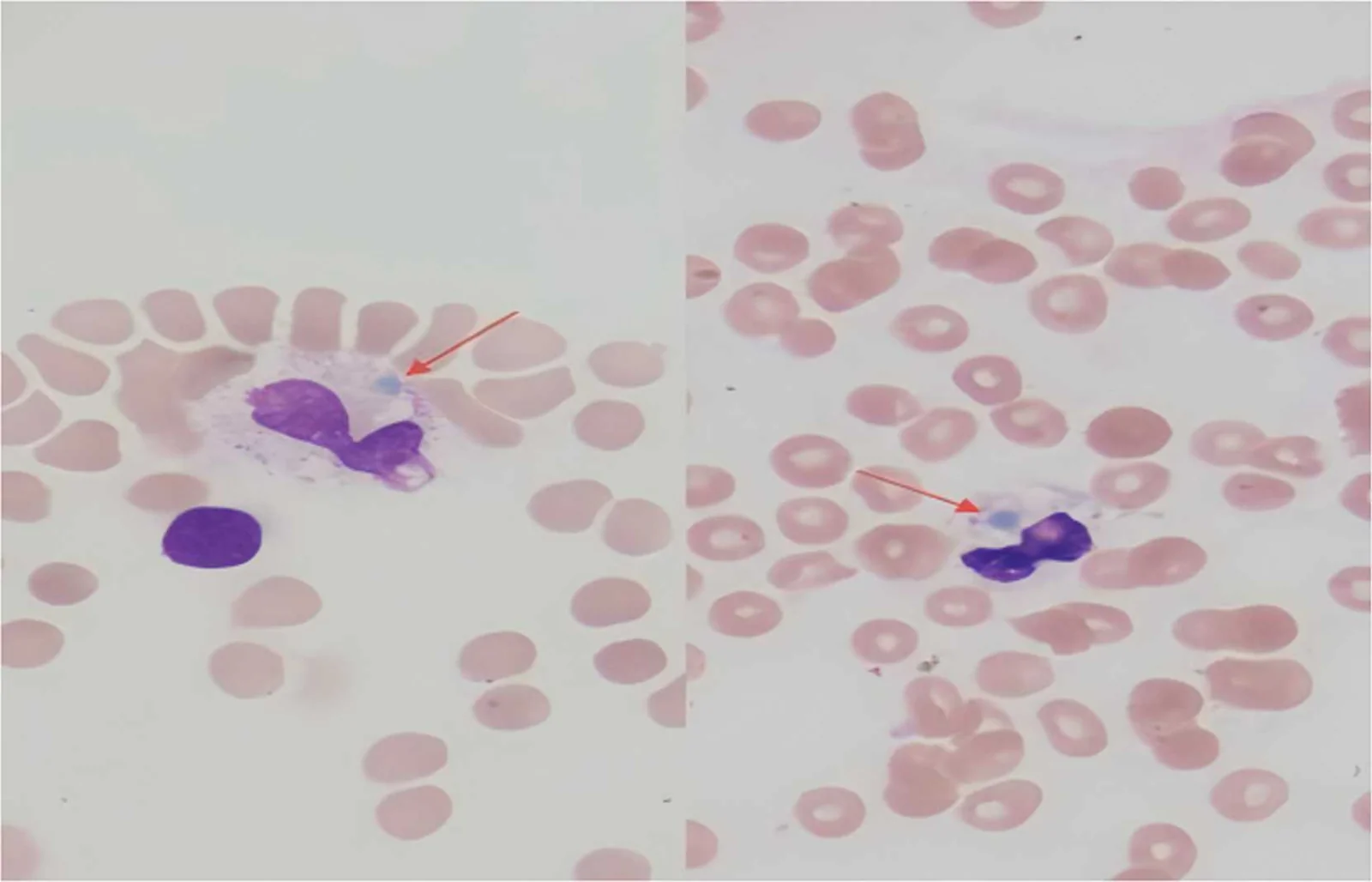
Wright-Giemsa stained peripheral blood (×1000) showing green bodies (arrow).

In 2023, Jiang W et al. [6] were the first to document the presence of green bodies in a patient diagnosed with Severe Fever with Thrombocytopenia Syndrome (SFTS). The morphology of the green bodies within neutrophils observed in our case (Fig. 3) aligns with the findings of this initial report. The presence of green bodies is indicative of a critical condition; however, this phenomenon is not exclusive to SFTS. The occurrence of green bodies within the neutrophil cytoplasm represents more than a mere morphological alteration; it serves as a significant indicator of severe hepatic dysfunction and is associated with an exceptionally high risk of mortality [7,8]. Both our case and the previously reported SFTS case involving green bodies [6,9] culminated in fatal outcomes.

**Fig. 4:**
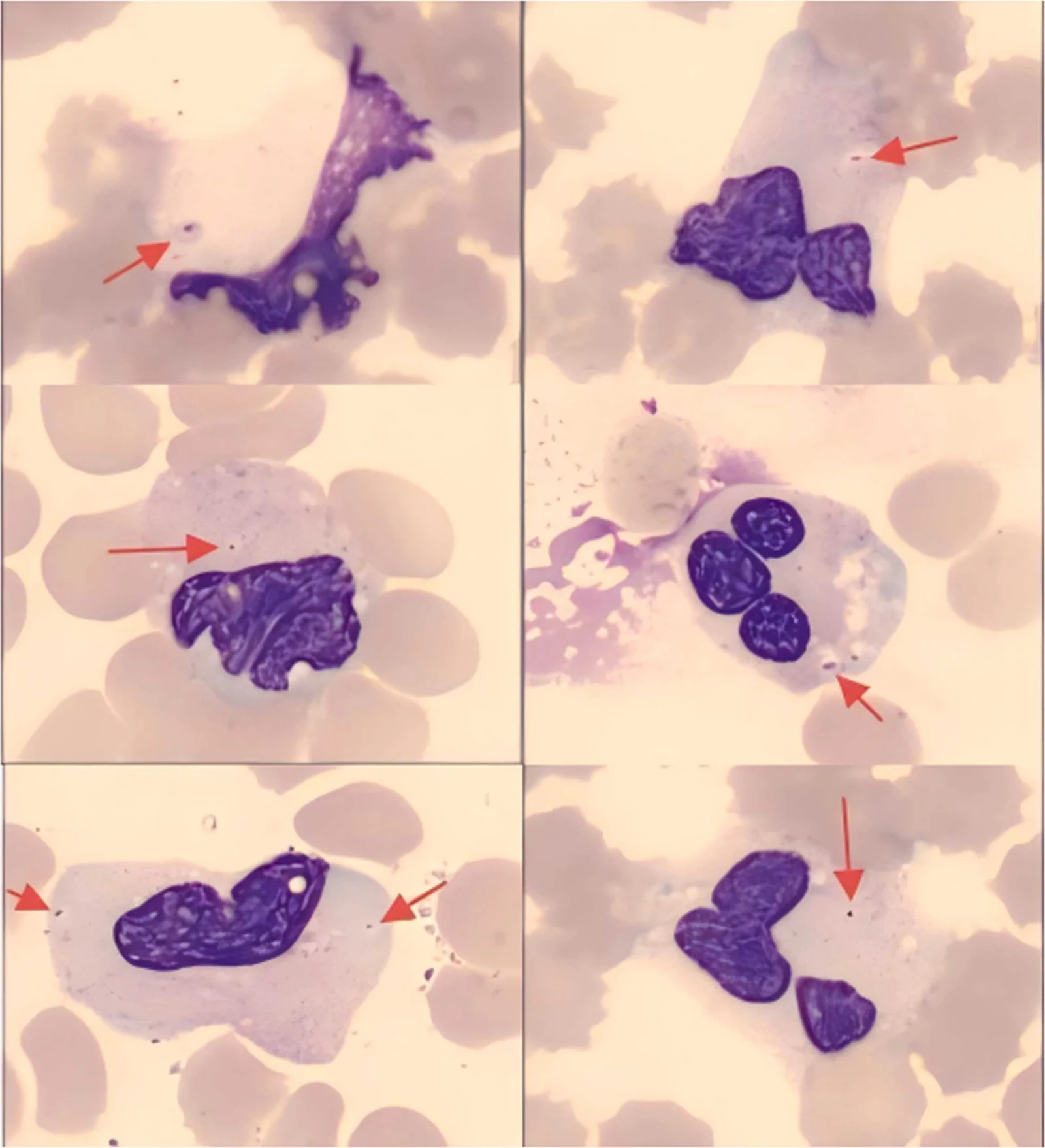
Wright-Giemsa stain of peripheral blood (×1000) showing phagocytized material in neutrophil cytoplasm (arrow).

Furthermore, we identified rare neutrophilic cytoplasmic inclusions (Fig. 4), which are distinct from the Howell–Jolly body-like inclusions described by Chen J et al. [9] in neutrophils of patients with severe fever with thrombocytopenia syndrome (SFTS). The inclusions observed in our study consist of fine phagocytosed granular particles within the cytoplasm, characterized by a clear halo separating the particles from the surrounding cytoplasm. These phagocytosed granules are notably different from both green bodies and Howell–Jolly bodies in terms of size and staining properties. To our knowledge, this specific type of neutrophilic cytoplasmic phagocytic granular inclusion has not been previously documented in the context of SFTS.

Moreover, the lymphocyte population included 6% plasmacytoid atypical lymphocytes (Fig. 2C–E). According to Huang M. et al. [10]fatal cases of severe fever with thrombocytopenia syndrome (SFTS) demonstrate elevated levels of plasmacytoid lymphocytes compared to survivors. The monocytes exhibited reactive changes, characterized by increased cell size, convoluted nuclei, and vacuolated cytoplasm (Fig. 2F). Significant thrombocytopenia was noted, along with the presence of giant platelets (Fig. 2G) and circulating apoptotic cells (Fig. 2H). Apoptotic cells may serve as potential indicators of high viral load, suggesting a poor prognosis [11]. These morphological observations in the peripheral blood of this patient align with those previously documented in SFTS patients in the literature [5,12]

This case, in conjunction with prior studies [5,13], corroborates four established morphological markers of Severe Fever with Thrombocytopenia Syndrome (SFTS): left shift, plasmacytoid lymphocytes, smudge cells, and apoptotic cells. Concurrently, it unveils several novel findings, such as the potential role of dysmorphic granulocytes as a primary source of smudge cells, the discovery of previously undocumented granular cytoplasmic inclusions within neutrophils, and the prognostic significance of green bodies and plasmacytoid lymphocytes. These detailed morphological characterizations fulfill a dual role: they assist in the early diagnosis of SFTS and facilitate timely prognostic evaluations. This dual functionality enhances prompt clinical decision-making and improves communication with patients' families regarding clinical expectations, thereby contributing to more effective patient management in regions where the disease is endemic.

## CRediT authorship contribution statement

**Junma You:** Writing – review & editing, Writing – original draft, Methodology, Investigation, Formal analysis, Conceptualization. **Zhiyong Feng:** Writing – original draft, Formal analysis, Conceptualization. **Hongsheng Zhang:** Validation, Supervision, Resources, Conceptualization.

## Ethics declaration

Written informed consent to take part in the study and to publish the article has been obtained from all participants or their legal representatives. The privacy rights of participants have been observed.

This study was performed in compliance with relevant laws, regulatory frameworks and guidelines where the research took place. The Medical Ethics Committee of Yichang Central People's Hospital granted an exemption. The authors provided the following explanation: This study is a single-case report involving the retrospective analysis of clinical and laboratory data from one patient. It does not meet the definition of a clinical trial or interventional research as per the relevant national regulations. The study strictly adheres to the principles of the Declaration of Helsinki and does not involve any experimental interventions or alterations to patient management. The informed consent for publication has been obtained from the patient's legal guardian. Due to its nature as a retrospective case report with no identifiable patient information beyond what is presented in the manuscript, and given that the research does not affect patient care or safety, this study is exempt from formal ethics committee review at our institution.

## Informed consent

Written informed consent was obtained from the patient's family for publication of this case report and accompanying images. A copy of the written consent is available for review by the Editor-in-Chief of this journal on request.

## Funding

The authors received no financial support for the research, authorship, and/or publication of this case report.

## Declaration of Competing Interest

The authors declare no conflicts of interest with respect to the research, authorship, and/or publication of this case report.

## Data Availability

The data supporting the findings of this case report are included within the article. Raw data are available from the corresponding author upon reasonable request.
